# Differential expression of coagulation pathway-related proteins in diabetic urine exosomes

**DOI:** 10.1186/s12933-023-01887-4

**Published:** 2023-06-22

**Authors:** Tianci Liu, Na Liu, Yizhao Wang, Tao Li, Man Zhang

**Affiliations:** 1 Clinical Laboratory Medicine, Beijing Shijitan Hospital, Capital Medical University, Beijing, 100038 China; 2 Beijing Key Laboratory of Urinary Cellular Molecular Diagnostics, Beijing, 100038 China; 3 Institute of Regenerative Medicine and Laboratory Technology Innovation, Qingdao University, Qingdao, 266071 China

**Keywords:** Diabetes mellitus, Urine exosomes, Coagulation-related proteins, F2, Biomarker

## Abstract

**Background:**

Coagulation function monitoring is important for the occurrence and development of diabetes. A total of 16 related proteins are involved in coagulation, but how these proteins change in diabetic urine exosomes is unclear. To explore the expression changes of coagulation-related proteins in urine exosomes and their possible roles in the pathogenesis of diabetes, we performed proteomic analysis and finally applied them to the noninvasive monitoring of diabetes.

**Methods:**

Subject urine samples were collected. LC-MS/MS was used to collect the information on coagulation-related proteins in urine exosomes. ELISA, mass spectrometry and western blotting were used to further verify the differential protein expression in urine exosomes. Correlations with clinical indicators were explored, and receiver operating characteristic (ROC) curves were drawn to evaluate the value of differential proteins in diabetes monitoring.

**Results:**

Analyzing urine exosome proteomics data, eight coagulation-related proteins were found in this study. Among them, F2 was elevated in urine exosomes of diabetic patients compared with healthy controls. The results of ELISA, mass spectrometry and western blotting further verified the changes in F2. Correlation analysis showed that the expression of urine exosome F2 was correlated with clinical lipid metabolism indexes, and the concentration of F2 was strongly positively correlated with blood TG levels (*P* < 0.05). ROC curve analysis showed that F2 protein in urine exosomes had a good monitoring value for diabetes.

**Conclusion:**

Coagulation-related proteins were expressed in urine exosomes. Among them, F2 was increased in diabetic urine exosomes and may be a potential biomarker for monitoring diabetic changes.

**Supplementary Information:**

The online version contains supplementary material available at 10.1186/s12933-023-01887-4.

## Introduction

Diabetes mellitus (DM) is a metabolic disease. It is mainly caused by insulin resistance or impaired insulin production and has reached epidemic proportions worldwide [1, 2]. At present, there are more than 537 million people with diabetes mellitus worldwide, and the number continues to rise [3]. Type II diabetes mellitus accounts for more than 90% of these cases, and the number of people with type II diabetes mellitus is expected to reach 700 million by 2045 [4], which is a serious threat to human life and health.

To date, diabetes mellitus remains incurable, and all patients with type I diabetes mellitus and advanced type II diabetes mellitus are treated with exogenous insulin [3]. As a chronic disease, diabetes mellitus needs lifelong monitoring. The current clinical indicators are still limited in detection. Long-term and repeated invasive blood drawing tests have caused physical and mental pain and burden to patients [5–7]. How to establish objective monitoring methods and minimize the trauma and mental burden of patients is one of the most challenging problems at present.

Urine is one of the most attractive samples for large-scale noninvasive clinical screening programs [8]. Urine is a potential source of disease biomarkers that could replace plasma. Exosomes are tiny membrane vesicles that are secreted by most cells in the body and range in diameter from 30 to 150 nm. Exosomes are derived from the endocytosis pathway of plasma membrane invagination and are a new library of biomarkers for discovery [9]. Exosomes are secreted by different cell types under normal and pathological conditions, and their contents and functions vary accordingly [10, 11]. Studies have found that exosomes are associated with pregnancy [12, 13], metabolism [14], cardiovascular disease [15], the central nervous system [16] and cancer progression [17]. In recent years, the continuous development of urine proteomics has provided room for the development of urine exosome protein biomarkers [18]. Although large clinical studies are required to validate these exosome biomarkers, the potential of urine exosomes as a noninvasive alternative to current diagnostic tests warrants further investigation.

Coagulation is the process in which coagulation factors are activated in a certain order to produce thrombin, and then converts fibrinogen into fibrin. The coagulation process needs to be finely regulated, and its coherence is of great importance [19]. Coagulation factors are mainly produced by the liver and circulate in the blood. Prothrombin, also known as factor II (F2), is the precursor of thrombin. Prothrombin is activated and hydrolyzed to thrombin by factor X and plays a key role in physiological and pathological coagulation processes [20]. Recent studies found that coagulation factors are also involved in tissue repair and inflammatory expression, including nervous system inflammation [20, 21].

As a chronic metabolic disease, diabetic patients often exhibit a hypercoagulable state. And hypercoagulability is an important factor for the occurrence and progression of diabetes. Studies have found that persistent elevation of blood glucose leads to prothrombin and fibrinogen glycosylation, which incompletely activates the coagulation cascade. At this point, the levels of many coagulation factors in the plasma increase, such as fibrinogen, factor VII, factor IX, factor XII, etc. [21, 22]. After that, the blood coagulation function and fat metabolism of the human changed significantly, which in turn promoted the occurrence and development of diabetes mellitus. Therefore, the monitoring of coagulation function has important clinical significance. It is also very important to understand the changes and influences of the expression of coagulation factors in the urine exosomes of diabetic patients to study the occurrence and development of diabetes mellitus.

The purpose of this study was to verify the expression of coagulation-related proteins in urine exosomes by means of proteomics and to explore the role of coagulation factors in the pathogenesis of diabetes mellitus. In addition, we evaluated its diagnostic and monitoring efficacy for diabetes mellitus, thereby providing suitable, sensitive, and specific biomarkers for diabetes monitoring.

## Materials and methods

### Patients

Patients with diabetes mellitus admitted to Beijing Shijitan Hospital from September 2020 to April 2021 were included as the study subjects, and healthy people with age and sex matching were selected as the healthy control. Diabetic patients had fasting blood glucose (FBG) ≥ 7.0 mmol/l, glycated hemoglobin (HbA1c) ≥ 6.5% or OGTT 2-hour blood glucose ≥ 11.1 mmol/L. The discovery cohort consisted of 30 patients with diabetes mellitus and 30 healthy controls. The test verification cohort consisted of 24 patients with diabetes mellitus and 24 healthy controls. And the validation cohort included 36 patients with diabetes mellitus and 36 healthy controls.

Clinical data and routine test indexes of subjects were collected retrospectively. All subjects were free of hematuria, proteinuria and ketosis. Urinary diseases and tumors were also excluded, and patients with a history of drug use for 2 weeks were excluded before urine samples were collected. All subjects provided informed consent prior to inclusion in the study and specimen collection. All procedures were carried out in accordance with the ethical standards of the Declaration of Helsinki and approved by the Ethics Committee of Beijing Shijitan Hospital.

### Exosome extraction

Thirty milliliters of clean morning urine was collected from the subjects, and the urine samples were centrifuged at 1500 g speed for 10 min and 10,000 g speed for 30 min to remove dead cells and debris. The exosomes were then collected using the size exclusion SEC method (qEV10/35 nm, IZON, Shanghai, China) [23–25]. Samples were stored at -80℃ until use. The morphology of exosomes was detected by transmission electron microscope (TEM); the size and concentration of exosomes were identified by nanoparticle tracking analysis (NTA); and the exosome markers and common exosome negative protein were analyzed by western blotting analysis.

### Mass spectrometry analysis of urine exosomes

Experiments were performed on a QExactive HF-X mass spectrometer (Thermo Fisher), and mass spectra were acquired in data independent acquisition (DIA) mode with a full scan range of m/z 350–1500 and resolution of 120,000 (m/z 200). Mass spectrometry results were queried in the SwissProt human database within UniProt (www.uniprot. org) using the proteome discovery software suite (Thermo Fisher Scientific v2.1). At the protein level, each protein contained at least one unique peptide using false discovery rate (FDR) of 1% as a filter. Proteins with a fold change > 1.5 and p value < 0.05 were considered significantly different.

### Western blotting

To further verify the proteomic analysis results of urine exosomes, we conducted western blotting experiments. After the exosomes were lysed, protein of equal mass was loaded onto a 12% Tris-HCl SDS-polyacrylamide gel. Transferred to PVDF membrane by Trans-Blot Turbo Transfer System (Bio-Rad, California, USA), and shaken for 2 h at room temperature after blocking with 5% skim milk in TBST. Then, the primary antibody (1:1000 dilution; Abcam, Cambridge, UK) was added and incubated overnight. Washed three times with TBST for 15 min each time, and added the horseradish peroxidase-labeled secondary antibody (diluted by 1: 2000; Bios, Beijing, China), and incubated at room temperature for 2 h. After that, they were washed three times with TBST and detected by enhanced chemiluminescence (ECL).

### Enzyme linked immunosorbent assay

The validation cohort included 36 patients with diabetes mellitus and 36 healthy controls who were age- and sex -matched. Urine exosomes were lysed with RIPA, and then the protein concentration was measured. The total mass of fixed protein was 10 µg, and the loading amount was adjusted to 100 µl with sample buffer. Then, a sample of 10 µl was added into each well using the YuanJu Biotechnology Center (Shanghai, China) ELISA kit. The concentration of target protein in exosomes was determined three times for each sample according to the instructions. The unknown sample concentration was calculated according to the standard curve, and the unit of target protein expression in exosomes was defined as pg/ml.

### Statistics

All experimental data were presented as the mean ± standard deviation (SD), and statistical analysis was performed by GraphPad Prism 8.0 (GraphPad, La Jolla, CA, USA) software. Student’s t-test was used for comparison of differences between groups. Pearson correlation was used for correlation analysis. The diagnostic performance of the target protein was evaluated using receiver operating characteristic curve (ROC) analysis. *P* < 0.05 was considered statistically significant.

## Results

### Clinical characteristics

The age and sex of all groups were matched, and the clinical data and relevant indicators of the selected subjects were shown in Table 1. There were no significant differences in aspartate aminotransferase (AST), alanine aminotransferase (ALT) and serum creatinine (Cr) between the diabetes mellitus patients and the healthy controls (*P* > 0.05).

**Table 1:** Clinical characteristics of diabetes mellitus patients (DM) and healthy controls (HC)

Characteristics	Discovery cohort			Test verification cohort				Validation cohort				
	NC (n = 30)	DM (n = 30)	*P*		NC (n = 24)	DM (n = 24)	*P*		NC (n = 36)	DM (n = 36)	*P*	
Age, years	53.73 ± 4.87	55.80 ± 5.06	ns		57.41 ± 5.78	55.92 ± 9.79	ns		52.44 ± 4.71	54.94 ± 6.30	ns	
TC, mmol/L	4.74 ± 0.88	4.27 ± 1.02	ns		4.51 ± 0.89	4.41 ± 1.07	ns		5.07 ± 1.02	4.76 ± 0.86	ns	
TG, mmol/L	1.23 ± 0.41	1.39 ± 0.33	ns		1.29 ± 0.44	1.53 ± 0.58	ns		1.60 ± 0.89	2.01 ± 0.90	ns	
LDL-C, mmol/L	3.12 ± 0.72	2.93 ± 0.92	ns		2.77 ± 0.84	3.08 ± 0.92	ns		3.49 ± 0.98	3.32 ± 0.70	ns	
UA, µmol/L	304.1 ± 65.08	339.6 ± 75.02	ns		318.1 ± 54.62	333.9 ± 98.65	ns		381.2 ± 85.10	361.8 ± 76.27	ns	
ALT, U/L	24.47 ± 7.99	23.03 ± 15.34	ns		19.79 ± 4.54	18.75 ± 6.28	ns		28.11 ± 15.17	29.83 ± 16.53	ns	
AST, U/L	21.43 ± 4.95	21.20 ± 8.79	ns		20.58 ± 2.88	23.45 ± 9.69	ns		22.11 ± 6.22	25.47 ± 16.83	ns	
Cr, µmol/L	77.33 ± 10.31	76.30 ± 20.09	ns		66.83 ± 11.13	62.08 ± 14.39	ns		78.53 ± 6.61	74.28 ± 12.91	ns	
ALB, g/L	44.14 ± 2.97	43.39 ± 2.32	ns		42.08 ± 2.11	40.62 ± 3.09	ns		44.49 ± 2.15	44.23 ± 2.12	ns	
FBG, mmol/L	5.53 ± 0.44	8.73 ± 1.91	***		5.25 ± 0.35	7.90 ± 2.62	***		5.39 ± 0.37	9.53 ± 2.45	***	
HbA1c, %	5.35 ± 0.50	7.62 ± 1.08	***		5.38 ± 0.20	8.87 ± 1.90	***		5.39 ± 0.32	7.91 ± 1.28	***	
UACR ratio, mg/g, n (%)												
< 30	30	30			24	24			36	36		
≥ 30	0	0			0	0			0	0		

Note: TC: Total Cholesterol; TG: Triacylglycerol; LDL-C: Low density lipoprotein cholesterol; UA: Uric acid; ALT: Alanine aminotransferase; AST: Aspartate aminotransferase; Cr: Creatinine; ALB: Albumin; FBG: Fasting Blood Glucose; HbA1c, glycated hemoglobin; UACR ratio, urinary albumin/creatinine ratio. P value: ns, no significance; ***, *P* < 0.001

### Characteristics of exosomes isolated from urine

**Fig. 1:**
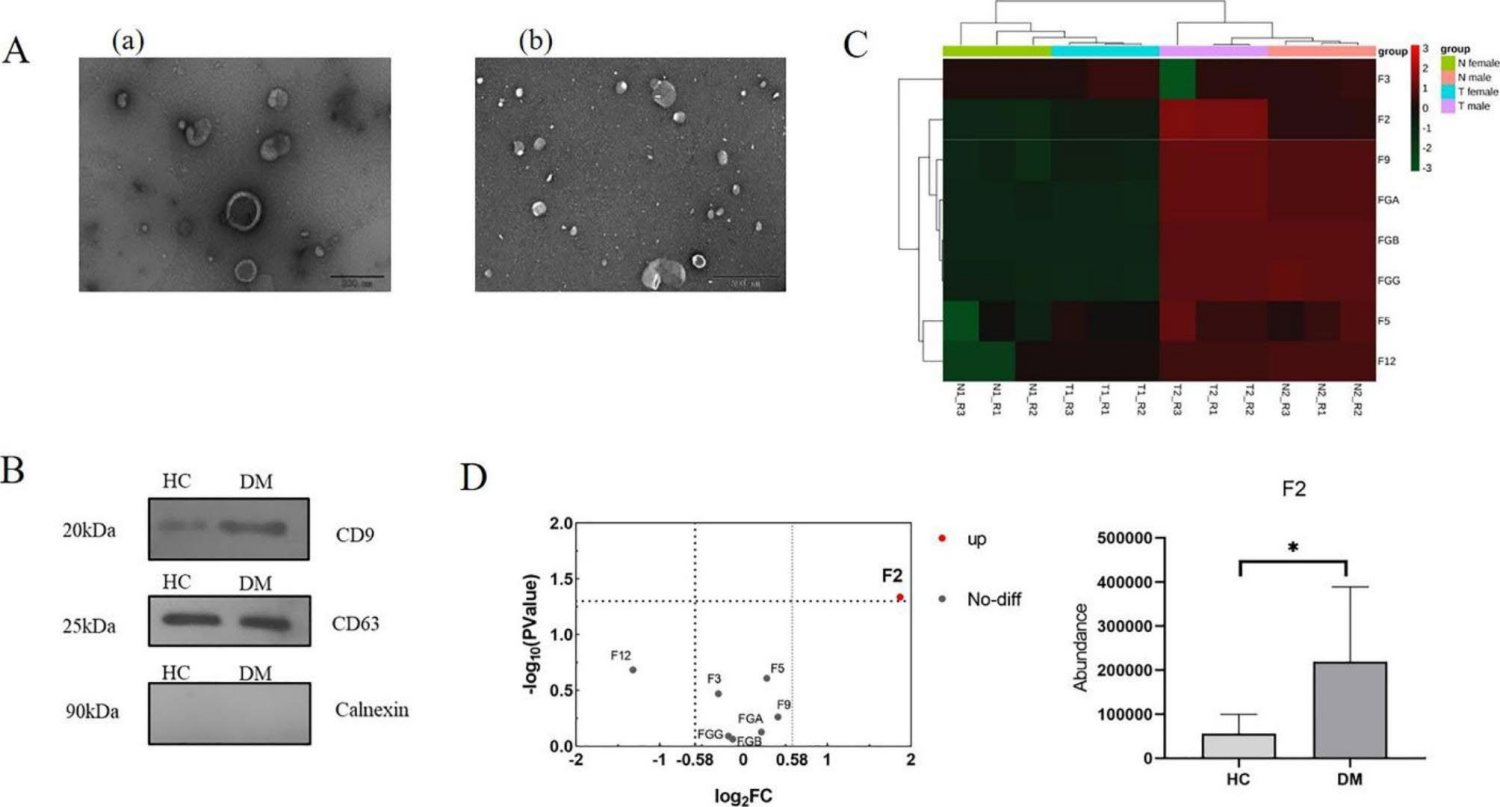
Identification of urine exosomes and proteomic analysis of coagulation-related proteins **A**: Representative TEM image of urine exosomes. Exosomes have an elliptic morphology (scale; a = 200 nm, b = 500 nm) **B**: Western blotting images of the markers and negative markers in the urine exosomes. Significant expression of CD9 and CD63 markers was observed in the healthy controls (HC) and diabetes mellitus patients (DM). Calnexin was not detected in the urine exosomes of samples **C**: Hierarchical clustering heatmap analysis of coagulation-related proteins in urine exosomes of the healthy controls (HC) and diabetes mellitus patients (DM). N1, females in HC group; N2, males in HC group; T1, females in DM group; T2, males in DM group **D**: Volcano plot analysis of coagulation-related proteins and relative abundance change of F2 protein in the healthy controls (HC) and diabetes mellitus patients (DM). The x-coordinate is denoted by log_2_ (FC) and the y-coordinate is denoted by -log_10_ (P), *, *P* < 0.05

Exosomes were isolated from the urine of samples, and the morphology of exosomes was detected by TEM. As shown in Fig. 1A, we observed an obvious double-membrane oval structure, and the size of exosomes ranged from 100 to 120 nm. Through NTA measurement, it was found that the average size of the exosomes was 123.7 nm (uploaded as Supplementary materials 1). Western blotting was used to detect the presence of two exosome markers: the transmembrane proteins CD9 and CD63 (Fig. 1B). As shown in the figure, exosomes isolated from the urine of the DM and HC groups expressed CD9 and CD63. Furthermore, all samples were negative for Calnexin, indicating fewer contaminants in the exosomes. According to the guidelines of the International Society of Extracellular Vesicles (ISEV) on the characterization of exosomes, our research results proved the existence of exosomes.

### Changes in the expression of coagulation-related proteins in urine exosomes

The false discovery rate (FDR) was set to 1%, and the MaxQuant human database was searched. According to the data of the discovery cohort, a total of eight coagulation-related proteins were identified in the urine exosomes (Supplementary material 2). The hierarchical clustering heatmap for these proteins was shown in Fig. 1C. Fold change > 1.5 and P value < 0.05 were considered significant differences. As shown in Fig. 1D, the expression of F2 protein in urine exosomes in diabetic patients was upregulated compared with that in healthy controls.

### Bioinformatics analysis of coagulation-related proteins in urine exosomes

**Fig. 2:**
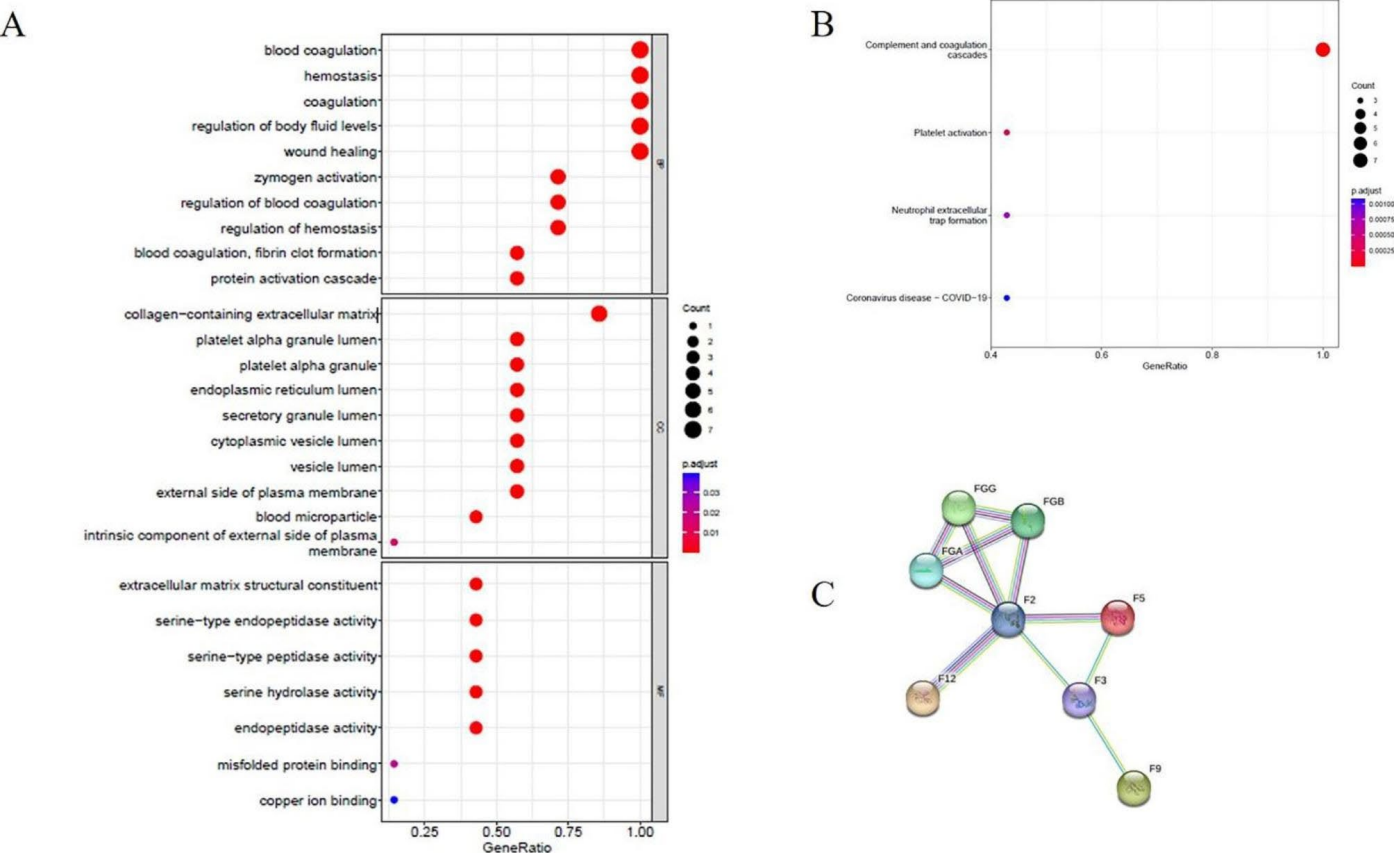
Bioinformatics analysis of coagulation-related proteins in urine exosomes **A**: GO enrichment analysis of 8 coagulation-related proteins. The ordinate represents GO functional categories: biological process (BP), cellular component (CC), and molecular function (MF). **B**: KEGG enrichment analysis of 8 coagulation-related proteins **C**: PPI network analysis of 8 coagulation-related proteins. More lines represent stronger correlations

We further performed GO and KEGG analysis of these 8 proteins, and the results were shown in Fig. 2. Biological process analysis showed that these proteins were closely related to blood coagulation, hemostasis, and regulation of body fluid levels. Most cellular components were located on the collagen-containing extracellular matrix, and their molecular function was mainly related to the extracellular matrix structural constituent (Fig. 2A). By KEGG pathway analysis, complement and coagulation cascades were the most enriched functions (Fig. 2B). At the same time, this study used the STRING database to predict the interactions of 8 different proteins. As shown in Fig. 2C, these proteins had strong interactions, of which the F2 protein was the core protein.

### Validation of coagulation-related protein expression in urine exosomes from diabetic patients

**Fig. 3:**
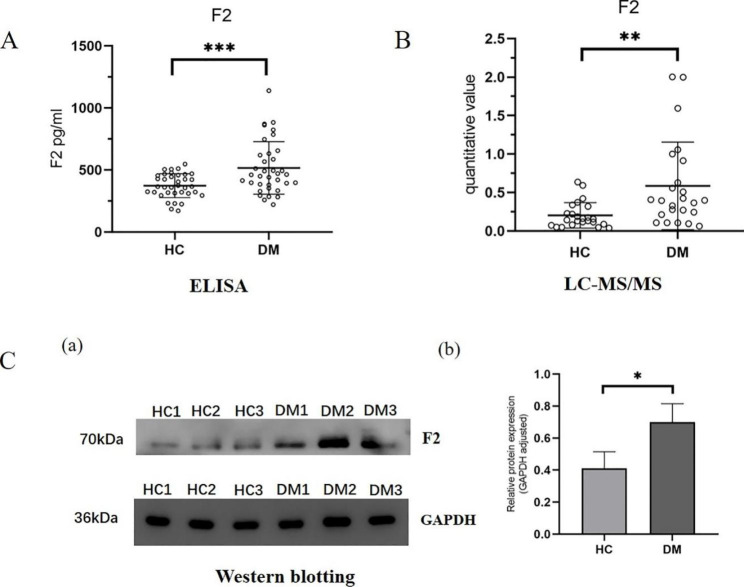
Validation of F2 protein expression in urine exosomes of diabetes mellitus patients and healthy controls **A**: Expression changes of urine exosome F2 in ELISA experiments of the validation cohort. The symbol represents individual subjects, and each subject is measured once in an independent experiment. ***, *P* < 0.001 **B**: Expression changes of urine exosome F2 in mass spectrometric of the test verification cohort. The symbol represents individual subjects, and each subject is measured once in an independent experiment. **, *P* < 0.01 **C**: (a) Western blotting image of urine exosome F2 and GAPDH proteins. The F2 protein was the target protein and the GAPDH protein was the internal reference protein (b) Gray scale values of F2 proteins after measurement with ImageJ. Adjusted with GAPDH. *, *P* < 0.05

We performed a series of validation experiments on differentially expressed F2 proteins in diabetic patients. According to the validation cohort, we conducted an ELISA experiment. The subjects included the diabetes mellitus patients (DM, n = 36) and the healthy controls (HC, n = 36), as shown in Fig. 3A. The expression level of F2 in urine exosomes of the diabetes mellitus patients was significantly higher than that in the healthy controls, and the difference was statistically significant (*P* < 0.05). According to the results of mass spectrometry data in the test verification cohort (Fig. 3B), the subjects included the diabetes mellitus patients (DM, n = 24) and the healthy controls (HC, n = 24). Compared with the healthy controls, the expression level of F2 in urine exosomes of the diabetes mellitus patients was significantly increased (*P* < 0.05). Urine exosomes from diabetes mellitus patients and healthy controls were selected for western blotting, as shown in Fig. 3C. F2 was the target protein and GAPDH was the internal reference protein in urine exosomes. In western blotting, F2 expression level was higher in urine exosomes of the diabetes mellitus patients compared with the healthy controls.

### Study on F2 protein expression in diabetic patients with different HbA1c levels

**Fig. 4:**
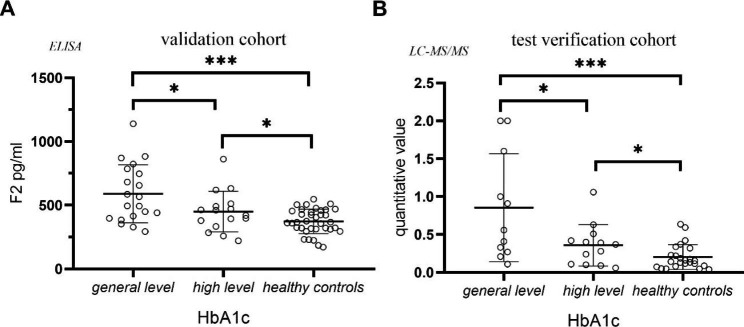
Study on F2 protein expression in diabetic patients under different HbA1c levels **A**: Expression changes of urine exosome F2 under different HbA1c levels in the validation cohort. The three groups included the general level group, high level group and healthy controls. The symbol represents individual subjects, and each subject is measured once in an independent experiment. ***, *P* < 0.001; *, *P* < 0.05 **B**: Expression changes of urine exosome F2 under different HbA1c levels in the test verification cohort. The three groups included the general level group, high level group and healthy controls. The symbol represents individual subjects, and each subject is measured once in an independent experiment. ***, *P* < 0.001; *, *P* < 0.05

Clinically, there is no clear standard for the classification of diabetic patients. In this experiment, we made a preliminary classification according to the HbA1c level. Patients with HbA1c < 8% were considered as general level, with good blood glucose control. Patients with HbA1c ≥ 8% were considered as high level, and blood glucose control was poor. According to the data of the validation cohort and test verification cohort, we explored the expression of F2 protein in different states of diabetes. As shown in Fig. 4, the expression of F2 in the urine exosomes of patients in the high level group was significantly lower than that in the general level group (*P* < 0.05), and the difference was statistically significant (*P* < 0.05). In addition, the expression of exosome F2 protein in two groups was higher than that in the healthy controls.

### Correlation analysis between the expression of F2 in urine exosomes and clinical indicators

**Fig. 5:**
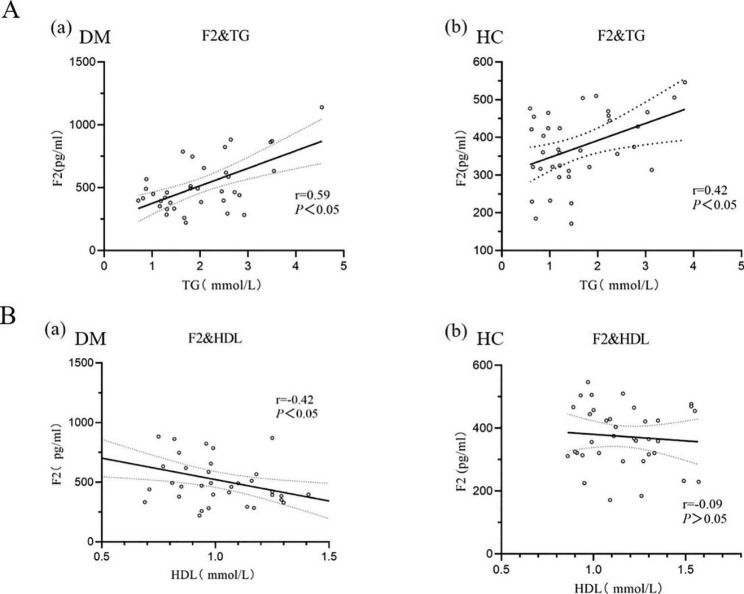
Correlation analysis of urine exosome F2 protein with TG and HDL in the diabetes mellitus patients (DM) and healthy controls (HC) **A**: (a) Correlation analysis between urine exosome F2 protein and TG in the DM (b) Correlation analysis between urine exosome F2 protein and TG in the HC. **B**: (a) Correlation analysis between urine exosome F2 protein and HDL in the DM (b) Correlation analysis between urine exosome F2 protein and HDL in the HC

Based on ELISA data from the validation cohort, we explored the correlation between F2 concentration in urine exosomes and clinical indicators, including TG, HDL-C, LDL-C, GLU, etc. The results were shown in Table 2. There was a strong positive correlation between urine exosome F2 concentration and blood TG. Urine exosome F2 was negatively correlated with HDL-C concentration and RBC number in diabetic patients, and positively correlated with basophil ratio, but there were no such correlations in normal subjects. Further investigation showed that the concentration of urine exosome protein F2 was linearly correlated with the blood TG level, as shown in Fig. 5. With the increase of F2 concentration in urine exosomes, the TG concentration may also increase.

**Table 2:** Correlation between urine exosome F2 concentration and clinical indicators

	HC		DM	
	r	*P*	r	*P*
TC	0.266	0.117	-0.258	0.128
TG	***0.420***	***0.011***	***0.594***	***0.001***
HDL-C	-0.090	0.602	***-0.415***	***0.011***
LDL-C	0.162	0.347	-0.243	0.154
GLU	0.125	0.469	0.081	0.640
TBIL	-0.312	0.064	-0.216	0.206
TP	0.042	0.808	-0.065	0.706
ALB	-0.097	0.574	0.082	0.633
ALT	-0.022	0.897	0.118	0.493
AST	0.041	0.813	-0.032	0.851
CRE	0.104	0.545	0.238	0.163
HbA1c	-0.017	0.920	-0.160	0.350
RBC	-0.065	0.706	***-0.331***	***0.049***
HGB	-0.124	0.470	-0.103	0.551
PLT	-0.083	0.630	-0.156	0.363
NE%	0.041	0.814	-0.254	0.134
LY%	-0.138	0.421	0.178	0.299
MO%	0.032	0.851	0.104	0.544
EO%	0.298	0.078	0.205	0.229
BA%	0.003	0.984	***0.518***	***0.001***

Bold italics represent correlations with P values less than 0.05

### Urine exosome F2 protein is of reference value for the auxiliary monitoring of diabetes mellitus

**Fig. 6:**
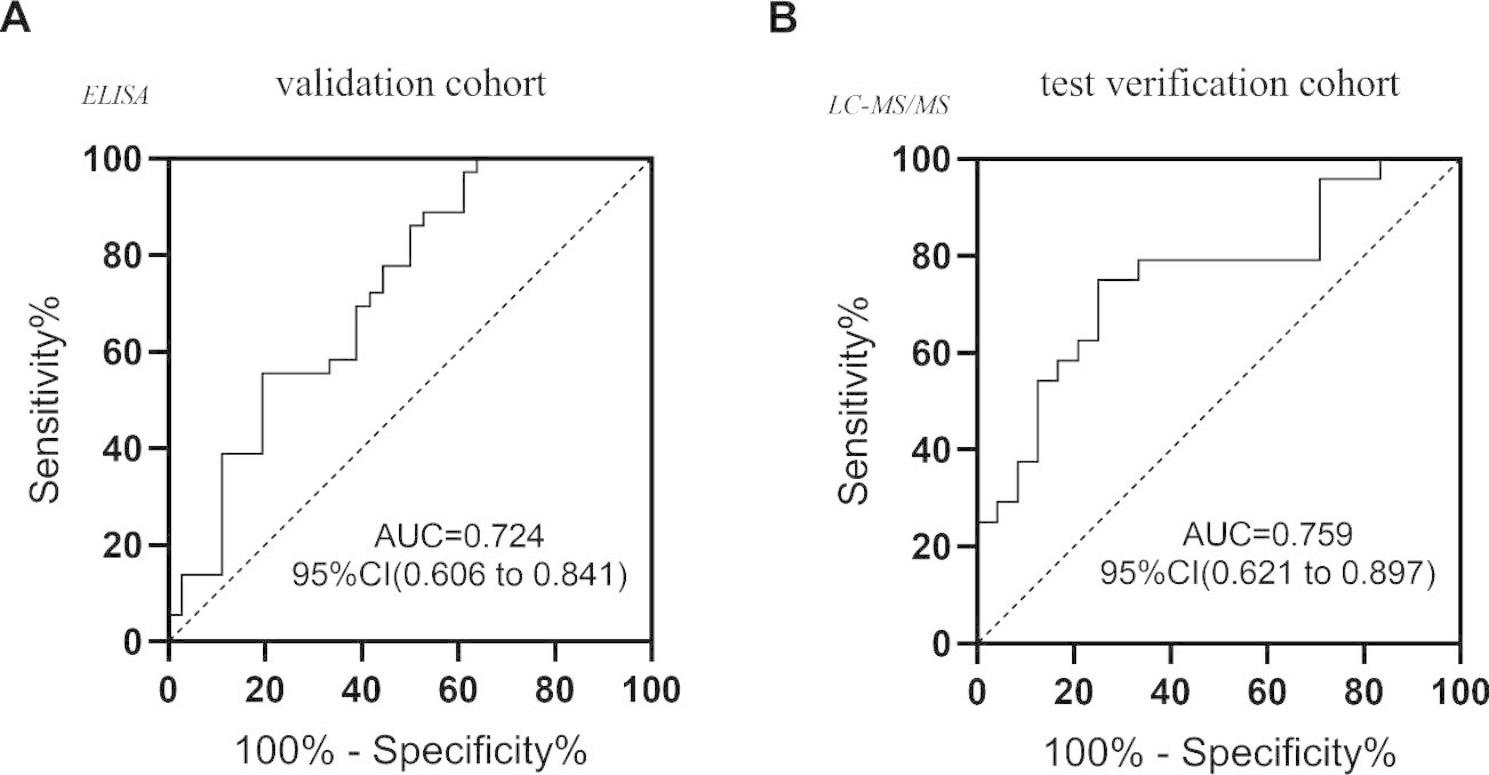
ROC curve analysis of urine exosome F2 protein in diabetic patients **A**: ROC curves were drawn based on ELISA data for diabetes mellitus patients (DM) and healthy controls (HC) in the validation cohort **B**: ROC curves were drawn based on mass spectrometry data for diabetes mellitus patients (DM) and healthy controls (HC) in the test verification cohort AUC, area under curve; CI, confidence interval

Based on the data of the test verification cohort and the validation cohort, the ROC curve was established to analyze the auxiliary monitoring value of F2 protein in urine exosomes, as shown in Fig. 6. The area under the F2 curve of urine exosomes in the validation cohort was 0.724 (95% confidence interval: 0.606 to 0.841). The area under the F2 curve of the urine exosomes in the test verification cohort was 0.759 (95% confidence interval: 0.621 to 0.897). In conclusion, urine exosome F2 has auxiliary diagnostic value and may be a potential biomarker for monitoring diabetes mellitus.

## Discussion

The coagulation is a continuous process, including the formation of prothrombin activator, thrombin formation, and fibrin production. Abnormalities at each stage can lead to disease. Patients with long-term diabetes mellitus greatly increase the risk of vascular diseases and microvascular complications, which seriously threaten the life and health of patients. It is of great significance to explore the expression changes of coagulation-related proteins in diabetic patients for the monitoring of diabetes mellitus and the control of diabetes complications.

In this study, a total of 8 coagulation-related proteins were identified in the urine exosomes of diabetic patients, and F2 protein with elevated expression was screened out. After that, ELISA, mass spectrometry, and western blotting were further used to verify the changes in F2 protein concentration in urine exosomes of diabetic patients, which were consistent with the protein changes observed in the discovery cohort. The F2 protein in urine exosomes can be used as a potential biomarker for monitoring diabetes mellitus and has clinical application value.

Coagulation factors are important proteins. The increase in F2 protein and thrombin production in the diabetes mellitus indicate that the blood of patients is in a state of hypercoagulation and that the activation of the coagulation system is enhanced. This is consistent with the results of previous studies [22]. Prothrombin is encoded by the F2 gene, which is located on chromosome 11 [26]. The gene contains 14 exons of 21 kb, and its structural integrity is essential for life and an important indicator for coagulation function monitoring. After vascular injury, prothrombin can be converted into active thrombin by prothrombinase. Thrombin is a macromolecular complex composed of factor Xa, factor Va, calcium ions, and phospholipids. Thrombin can convert fibrinogen to fibrin, activate platelets and increase endothelial permeability, thereby preventing blood loss at the site of injury and promoting vascular remodeling [27–29]. The integrity of the prothrombin structure is essential for life, and mice lacking prothrombin die prematurely in the early embryonic stage [30].

Diabetes mellitus is a hypercoagulable state [31], especially in patients with uncontrolled diabetes mellitus. Glycemic control is one of the important elements to assess in patients with diabetes mellitus. In this study, the level of F2 expression in the urine exosomes of patients at a high level was significantly lower than that of patients at a general level. For diabetic patients with HbA1c ≥ 8%, blood glucose control was not ideal, and F2 expression was lower. This may be because when the blood glucose control of diabetic patients worsens, the increase in plasma glucose and insulin leads to a rapid and substantial increase in the circulation of thrombin production [32]. In this study, the level of F2 expression in the urine exosomes of patients at a general level was significantly higher than that of the normal controls. Meanwhile, when we removed the data of the high level group and reanalyzed the area under the curve of F2 protein, as shown in Figure S1, we found that the area under the curve of F2 protein is larger at this time. It is speculated that urine exosome protein F2 may have higher clinical value for the early diagnosis of diabetes mellitus. In conclusion, F2 can be used as a monitoring indicator of diabetes mellitus, especially for blood glucose control monitoring, which has very important clinical value.

Studies have shown that cholesterol plays a crucial role in determining cell membrane properties and function, and the accumulation of cholesterol in pancreatic β cells can lead to cell dysfunction [33]. High levels of TG and LDL and low levels of HDL are associated with insulin resistance and are independent factors in insulin development [34]. Elevated blood glucose occurs when the function of pancreatic β cells in the body to increase insulin release fails to compensate for the degree of insulin resistance. In vitro experiments have shown that HDL increases skeletal muscle glucose uptake and stimulates pancreatic β cells to synthesize and secrete insulin [35, 36]. Hypertension can aggravate insulin resistance, thus exacerbating the occurrence and development of diabetes. In this study, we found a strong positive correlation between urine exosome F2 and TG concentration in blood through exploration. F2 and TG concentrations are increased in diabetic patients, which may be one of the reasons why diabetic patients are more likely to be complicated with vascular lesions. In addition, we found that the F2 protein concentration was not associated with blood HDL-C in the healthy controls but was strongly negatively associated with blood HDL-C in the diabetes mellitus group. This correlation gives us a guess about the F2 protein. With the disorder of glucose metabolism in the human body, the concentration of F2 protein increases, which will affect the expression of HDL-C. Low levels of HDL-C cause insulin resistance, which in turn contributes to the development and progression of diabetes mellitus. The rise in blood sugar and insulin disorder further lead to abnormal changes in blood lipids [37]. Of course, at present, this is just our guess, and the specific reason needs to be further studied in the molecular mechanism.

This study is the first to report the expression of coagulation-related proteins in urine exosomes of diabetic patients. In ROC curve analysis, we found that urine exosome protein F2 has good diagnostic ability and could be a potential biomarker for monitoring diabetic changes. Early detection of individuals at risk of developing diabetes mellitus may benefit for the implementation of preventive treatment. Urine samples have unique advantages in disease screening. Urine exosomes can meet the need for novel markers of diabetes mellitus and can be used for noninvasive, rapid and simple outcome determination [38].

The biomarker found in this study needs to be further evaluated and verified by many samples before being used in clinical practice. In the future, this study will be applied to the clinical monitoring of diabetes mellitus and could benefit a large population.

## Conclusions

In summary, this study investigated the expression of coagulation-related proteins, especially F2 protein, in urine exosomes of diabetic patients by mass spectrometry. The expression change of F2 is associated with lipid metabolism and may serve as a potential biomarker for monitoring diabetic changes, providing a promising approach for the noninvasive diagnosis and monitoring of diabetes mellitus.

## Electronic supplementary material

Below is the link to the electronic supplementary material.

Supplementary Material 1: Representative NTA results of urine exosomes.

Supplementary Material 2: Expression of coagulation-related proteins in urine exosomes.

Supplementary Material 3. Figure S1. ROC curve analysis of urine exosome F2 protein after removing the high level group. A: ROC curves were drawn based on ELISA data for general level group and healthy controls (HC) in the validation cohort. B: ROC curves were drawn based on mass spectrometry data for general level group and healthy controls (HC) in the test verification cohort. AUC, area under curve; CI, confidence interval.

## Data Availability

The datasets generated and analysed during the current study are not publicly available but are available from the corresponding author on reasonable request.
