# Supplementary material for: Differential expression of coagulation pathway-related proteins in diabetic urine exosomes
Source: Cardiovasc Diabetol. 2023 Jun 22;22:145. doi: 10.1186/s12933-023-01887-4 (PMC10288686; doi:10.1186/s12933-023-01887-4)
Supplement: Supplementary file 1 — Supplementary Material 1: Representative NTA results of urine exosomes. [file 12933_2023_1887_MOESM1_ESM.pdf]

ZetaView® Laser Scattering Video Microscope

PARTICLE METRIX
www.particle-metrix.de

Electrophoresis & Brownian Motion
Video Analysis
Laser Scattering Microscopy

Operator (Report): Administrator
Video Operator: Administrator

Sample Parameters
Sample Name: 196
Comment: Au20nm 1:5000, Sample Remarks0:
Sample Remarks1:
Sample Remarks2:
Electrolyte: h2o
Temperature: 27.95 °C sensed
pH 7.4 entered
Conductivity: 0.00 µS/cm entered

Result (sizes in nm)

	Number	Concentration	Volume
Median (X50)	119.1	119.1	189.1
Span	55.8	55.8	79.5

Concentration: 2.0E+8 Particles / mL
Dilution Factor: 100
Original Concentration: 2.0E+10 Particles / mL

Quality
Average Counted Particles per Frame: 444
Number of Traced Particles: 1031

Measurement Parameters
Cell S/N: NTA

Measurement Mode: Size Distribution 4 Cycles
11 Positions, 2 Removed for Analysis

Analysis Parameters
Max Area: 1000, Min Area: 10, Min Brightness: 30

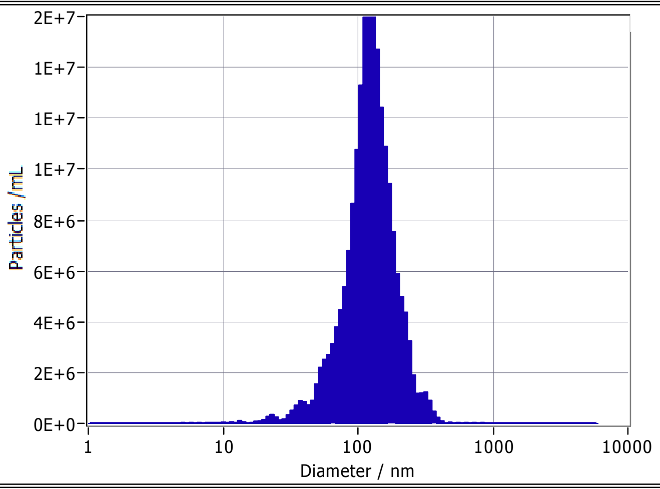

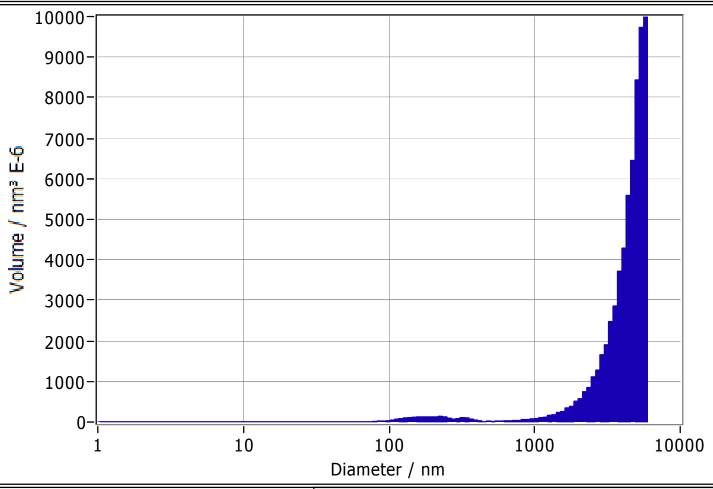

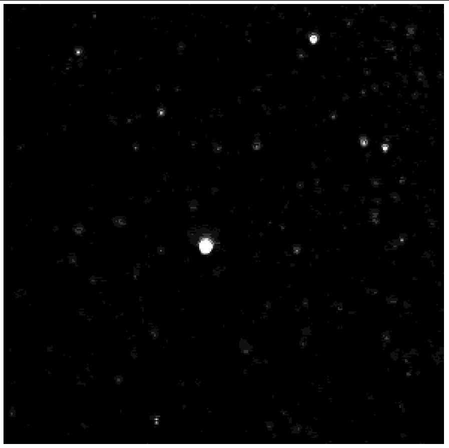

Peak Analysis (Concentration)

Diameter / nm	Particles/mL	FWHM / nm	Percentage
123.7	1.7E+7	82.9	98.9
13.1	9.9E+4	2.5	0.1
557.1	3.9E+4	119.0	0.1
9.5	3.5E+4	4.6	0.1
7.0	3.3E+4	1.2	0.1

X Values

	Number	Concentration	Volume
X10	67.1	67.1	115.1
X50	119.1	119.1	189.1
X90	194.6	194.6	318.4
Span	1.1	1.1	1.1
Mean	131.6	131.6	210.4
StdDev	55.8	55.8	79.5

Comment

(Signature)

Analyzed Video: E:\SXD-NTA\20201224\tmz-u87-zjw-exo-2\20220321_0002_dpmsc1_size.avi

ZetaVIEW S/N 17-340, Software ZetaView 8.04.02, Camera 0.714 µm/px

Experiment: 2022-03-21 19:43, Report: 2022-03-21 20:08
