# Supplementary material for: Differential expression of coagulation pathway-related proteins in diabetic urine exosomes
Source: Cardiovasc Diabetol. 2023 Jun 22;22:145. doi: 10.1186/s12933-023-01887-4 (PMC10288686; doi:10.1186/s12933-023-01887-4)
Supplement: Supplementary file 2 — Supplementary Material 2: Expression of coagulation-related proteins in urine exosomes. [file 12933_2023_1887_MOESM2_ESM.docx]

***Supplementary material 2*** Target proteins in urine exosomes

| **UniProt-ID** | **Protein Name** | **Gene Name** | **FC** | **P-value** | **Form of expression** |
| --- | --- | --- | --- | --- | --- |
| P00734 | Prothrombin | F2 | 3.65 | 4.59E-02 | up |
| P00748 | Coagulation factor XII | F12 | 0.40 | 2.07E-01 | ns |
| P00740 | Coagulation factor IX | F9 | 1.33 | 5.46E-01 | ns |
| P12259 | Coagulation factor V | F5 | 1.21 | 2.46E-01 | ns |
| P13726 | Tissue factor | F3 | 0.81 | 3.38E-01 | ns |
| P02671 | Fibrinogen alpha chain | FGA | 1.16 | 7.45E-01 | ns |
| P02675 | Fibrinogen beta chain | FGB | 0.91 | 8.64E-01 | ns |
| P02679 | Fibrinogen gamma chain | FGG | 0.88 | 8.13E-01 | ns |
