# Supplementary figures and images for: Differential expression of coagulation pathway-related proteins in diabetic urine exosomes
Source: Cardiovasc Diabetol. 2023 Jun 22;22:145. doi: 10.1186/s12933-023-01887-4 (PMC10288686; doi:10.1186/s12933-023-01887-4)

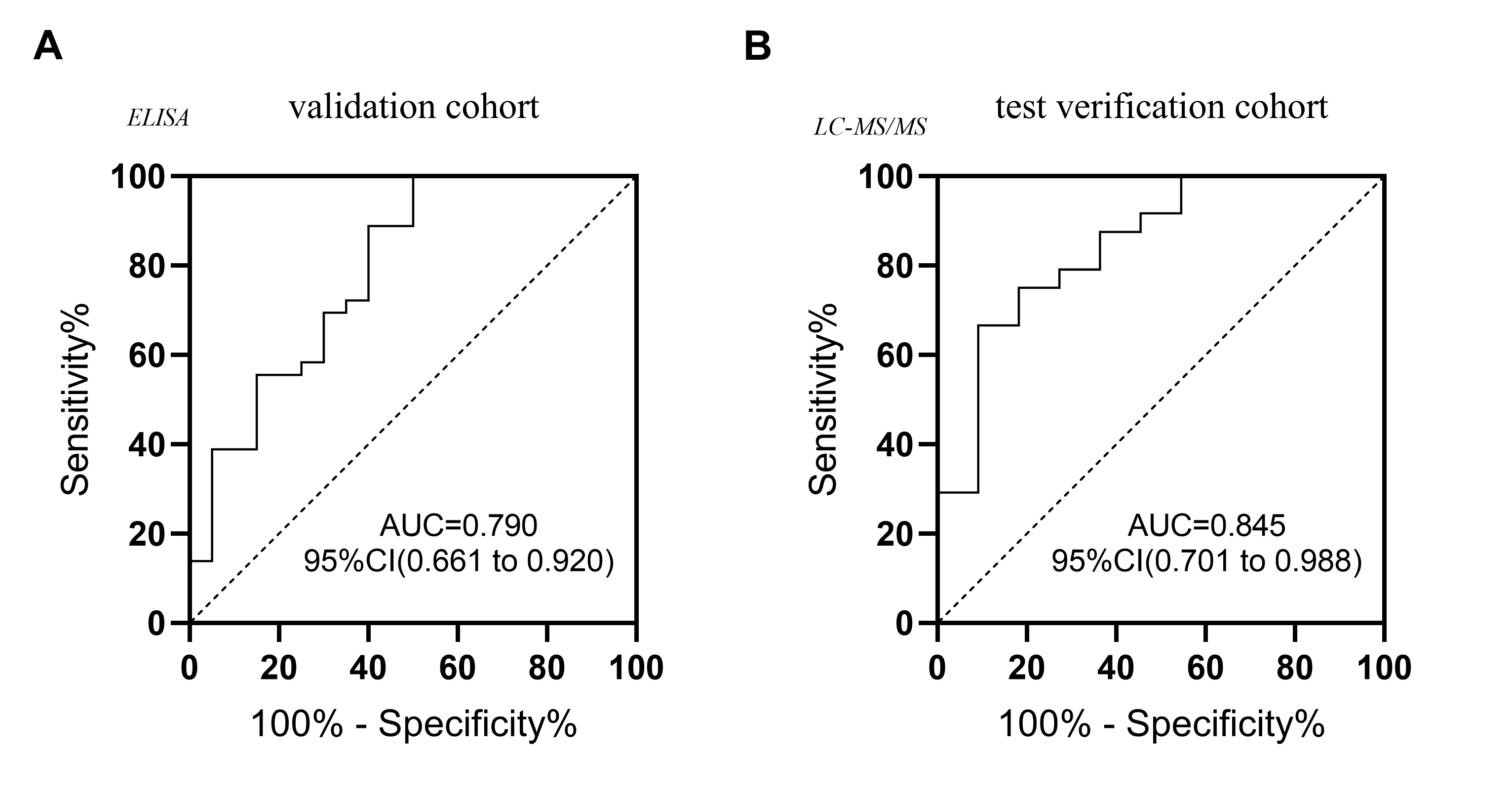

Supplement: Supplementary file 3 — Supplementary Material 3. Figure S1. ROC curve analysis of urine exosome F2 protein after removing the high level group. A: ROC curves were drawn based on ELISA data for general level group and healthy controls (HC) in the validation cohort. B: ROC curves were drawn based on mass spectrometry data for general level group and healthy controls (HC) in the test verification cohort. AUC, area under curve; CI, confidence interval. [file 12933_2023_1887_MOESM3_ESM.tif]
